# A pilot randomized controlled trial to compare the effectiveness of two 14-day primaquine regimens for the radical cure of vivax malaria in South India

**DOI:** 10.1186/s12936-018-2472-5

**Published:** 2018-09-03

**Authors:** Kavitha Saravu, Chaitanya Tellapragada, Shrivathsa Kulavalli, Wilbin Xavier, Shashikiran Umakanth, Gouthami Brahmarouphu, Navyasree Kola Srinivas, Jagadish Puralae Channabasavaiah, Anzil Bava, Abdul Vahab Saadi, Vasudev Guddattu, Kapaettu Satyamoorthy, Krishnamurthy Bhat

**Affiliations:** 1 Department of Medicine, Kasturba Medical College, Manipal Academy of Higher Education, Manipal, Madhava Nagar, Manipal, Karnataka 576104 India; 2 Manipal McGill Centre for Infectious Diseases, Prasanna School of Public Health, Manipal Academy of Higher Education, Madhava Nagar, Manipal, Karnataka 576104 India; 3 Department of Virus Research, Manipal Academy of Higher Education, Madhava Nagar, Manipal, Karnataka 576104 India; 4 Department of Pharmaceutical Quality Assurance, Manipal College of Pharmaceutical Sciences, Manipal Academy of Higher Education, Madhava Nagar, Manipal, Karnataka 576104 India; 5 Department of Cell & Molecular Biology, School of Life Sciences, Manipal Academy of Higher Education, Madhava Nagar, Manipal, Karnataka 576104 India; 6 Department of Statistics, Manipal Academy of Higher Education, Madhava Nagar, Manipal, Karnataka 576104 India; 7 Department of Medicine, Dr. TMA Pai Hospital, Udupi, Melaka Manipal Medical College, Manipal Academy of Higher Education, Madhava Nagar, Manipal, Karnataka 576104 India

**Keywords:** *Plasmodium vivax*, Recurrent malaria, Primaquine, Carboxyprimaquine, Relapse, RP-HPLC, India

## Abstract

**Background:**

Radical cure of *Plasmodium vivax* malaria requires treatment with a blood schizonticide and a hypnozoitocide (primaquine) to eradicate the dormant liver stages. There has been uncertainty about the operational effectiveness and optimum dosing of the currently recommended 14-day primaquine (PQ) course.

**Methods:**

A two centre, randomized, open-label, two arm study was conducted in South India. Patients were randomized to receive either high dose (0.5 mg base/kg body weight) or conventional dose (0.25 mg/kg) PQ for 14 days. Plasma concentrations of PQ and carboxyprimaquine (CPQ) on the 7th day of treatment were measured by reverse phase high performance liquid chromatography. Study subjects were followed up for 6 months. Recurrent infections were genotyped using capillary fragment length polymorphism of two PCR-amplified microsatellite markers (MS07 and MS 10).

**Results:**

Fifty patients were enrolled. Baseline characteristics and laboratory features did not differ significantly between the groups. Mean age of the study population was 42 ± 16.0 years. Recurrences 80–105 days later occurred in 4 (8%) patients, two in each the groups. All recurrences had the same microsatellite genotype as that causing the index infection suggesting all were relapses. One relapse was associated with low CPQ concentrations suggesting poor adherence.

**Conclusions:**

This small pilot trial supports the effectiveness of the currently recommended lower dose (0.25 mg/kg/day) 14 day PQ regimen for the radical cure of vivax malaria in South India.

*Trial registration* Clinical Trials Registry-India, CTRI/2017/03/007999. Registered 3 March 2017, http://ctri.nic.in/Clinicaltrials/regtrial.php?modid=1&compid=19&EncHid=82755.86366.

## Background

An estimated 2.5 billion people live in areas where *Plasmodium vivax* infection is endemic [1]. India, Ethiopia, Indonesia and Pakistan account for > 80% of the world’s *vivax* malaria [2]. Malaria remains a major public health concern in India where epidemiology varies substantially across the country [3]. Over the past decade, deployment of rapid diagnostic tests, allowing early diagnosis and treatment, and aggressive vector control measures have both helped to reduce malaria case fatality rates significantly. As mortality from falciparum malaria has declined the incidence of *P. vivax* in some states of India has increased. Approximately 12% of these cases occur in the urban areas [3]. *Plasmodium vivax* presents a substantial challenge for malaria control and elimination targets because of relapse, resulting from activation of dormant liver stages (hypnozoites) [4]. Prevention of relapse requires use of 8-aminoquinoline anti-malarials, in addition to schizontocidal treatment of the blood stage infection, with attendant risks of haemolysis in patients who have glucose-6-phosphate dehydrogenase (G6PD) deficiency [5]. Although PQ radical cure is widely recommended it is often not prescribed because G6PD testing is seldom available.

Several different factors determine the radical curative efficacy of PQ. These include the dosage administered, duration of treatment, host genetic polymorphisms in cytochrome P450 metabolizing enzymes (notably 2D6), and parasite susceptibility [6]. Adherence to the currently recommended 14-day PQ regimens is often regarded as poor, although the evidence is conflicting [6, 7]. The Indian National Vector Borne Disease Programme (NVBDP) recommends radical cure regimen comprising 0.25 mg/kg body weight for 14 days (http://nvbdcp.gov.in/Doc/National-Drug-Policy-2013.pdf) [8]. In contrast, the Centers for Disease Control and Prevention (CDC), Atlanta, recommends a higher dose regimen (0.50 mg/kg body weight for 14 days) [9]. WHO recommends the higher dose PQ regimen for areas with frequent relapse *P. vivax* strains such as East Asia [12]. There is, therefore, some uncertainty over the optimum dose and the effectiveness of currently recommended regimens. This prospective study compared and evaluated the effectiveness of two radical cure regimens in the treatment of vivax malaria in the South of India.

## Methods

### Study population

Patients with acute vivax malaria attending adult medical units at Kasturba Hospital, Manipal and Dr T M A Pai Hospital, Udupi, India were included in the study, if they met the following inclusion criteria: adults of either gender (≥ 18 years old) with *P. vivax* mono-infection confirmed by microscopy, fever defined as > 37.5 °C tympanic or oral temperature or a history of fever within previous 3 days, and willing to give informed consent. Pregnant and/or lactating women, and patients with G6PD deficiency or mixed infection with *P. vivax* and *Plasmodium falciparum* were excluded.

### Study design

This prospective, two-centre, randomized, open-label, two-arm pilot study, enrolled patients from March to August 2017. In this pilot study, it was planned to enroll at least 25 patients in each arm.

### Study setting

Patients were diagnosed with vivax malaria at Kasturba Hospital, Manipal and Dr. Pai Hospital, Udupi, tertiary and secondary care hospitals, respectively, in Udupi district of Karnataka State, India. Udupi district is located 13°32′24.43″ N latitude and 74°52′26.78″ E longitude, with typical tropical climatic conditions. The monsoon period falls June to October, with average rainfall of more than 4000 mm every year. Malaria incidence occurs throughout the year in Udupi district with peaks around June to July [10]. The mean annual parasite incidence was estimated as 2.5 per 1000 risk population during years 2012 to 2014 [11]. The catchment area of Kasturba Hospital, Manipal encompasses both the rural and urban population of coastal and interior Karnataka, Goa and Kerala. Kasturba Hospital, Manipal and Dr Pai Hospital, Udupi, record malaria incidence across the year with over 200 malaria cases per annum, each comprising > 55% *P. vivax* cases.

### Subject recruitment and data collection

Eligible subjects were recruited in the study after obtaining written informed consent. A structured proforma was used to collect data. Data regarding history of fever, headache, vomiting, and past history of malaria, as well as patient’s nutritional status, socio-demographic information and general information on diabetes, hypertension, chronic kidney disease, and HIV were collected. Laboratory investigations included haemoglobin, haematocrit, platelet count, erythrocyte sedimentation rate, G6PD levels, urea, creatinine) and schizonticidal treatment [whether chloroquine (CQ) or artemisinin-based combination therapy (ACT)], hypnozoitocidal drug- PQ regimen along with its initiation date. Cases were classified into severe and non-severe cases as per WHO severity criteria [12]. The subjects’ follow-up details were also documented, and all the collected data were double-entered using an electronic clinical record form (SPSS 15).

### Subject allocation and intervention

After completion of treatment of acute malaria with a schizonticidal drug [either chloroquine 25 mg base/kg or ACT (astesunate with doxycycline OR artemether-lumefantrine) as per the treating clinician’s judgement of severity], patients were randomized into the study. A block randomization was used to allocate subjects to the groups. Five blocks of size 10 were used. The randomization sequence within each block was done using a lottery method. Eligible patients were allocated to one of the following two treatment groups.

1. Primaquine 0.5 mg base/kg/day for 14 days (IPCA Laboratory Pvt Ltd, Mumbai).
2. Primaquine 0.25 mg base/kg/day for 14 days (IPCA Laboratory Pvt Ltd, Mumbai).

The subjects were advised to take the drug once a day in the morning after breakfast. Drug dosing was not observed. They were counselled about the importance of PQ for the effective cure of malaria and adherence to medication. Additionally, patients were counselled about personal protective measures against mosquito bite.

### Subject follow-up

The study subjects were followed up on day 7 of PQ treatment for PQ and CPQ estimation. Patients were asked to come for blood draw, 2 h after the intake of PQ, in a fed state. Self-reported time after intake of PQ to blood collection was noted. Three ml of venous plasma was collected in EDTA vacutainer for drug estimation. Participants were questioned about compliance to study medication, and confirmed by used blister packs of PQ.

Any adverse events reported by the subject were noted. Patients were encouraged to return if ill. During the follow-up period, peripheral blood smears for parasitic forms were examined on day 28 from onset of illness, followed by a periodic examination by the end of every second month until the completion of 6 months, and if any fever episodes occurred in the follow-up period, the subjects’ blood smears were examined for parasitic forms. Treatment adherence, mosquito repellent and bed net usage, intercurrent febrile illnesses and any other possible symptoms of malaria were recorded and documented. In cases with microscopy confirmed recurrence of malaria, patients were treated with CQ/ACT as per the severity of illness followed by PQ 0.5 mg/kg/day for 14 days.

### Genetic characterization of *Plasmodium vivax* for differentiating relapse from re-infection

During the initial recruitment, 2 ml of blood sample was collected from all study subjects in heparinized vacutainers and preserved in frozen conditions until further testing. Two ml of heparinized blood was also collected from patients diagnosed (smear positive) with recurrence of malaria. DNA was extracted from the heparinized blood specimens of patients (n = 4) collected during initial and recurrent infections of malaria. For DNA extraction, HiPurA™ Blood Genomic DNA Mini Purification kit meant for DNA isolation from fresh or frozen blood (HiMedia Laboratories, India) was used as per the manufacturer’s instructions.

Genotyping of *P. vivax* was performed based on sequence repeats in the microsatellites markers MS7 and MS10 using primers and the method described by Karunaweera et al. [13]. The microsatellite markers were amplified by PCR using 3 µl of extracted DNA with a PCR reaction performed in a final volume of 20 µl containing 1× PCR buffer, 2 mM of MgCl_2_, 200 mM of each dNTP, 0.25 mM of each primer, and 1.5 units of Taq DNA polymerase (Invitrogen). The following thermal cycling conditions were maintained: 5 min at 95 °C followed by 30 cycles of denaturation for 45 s at 95 °C, annealing for 45 s at 56 °C and 62 °C (for MS7 and MS10 markers, respectively), and elongation for 1 min at 72 °C with a final elongation for 5 min at 72 °C. Primers for markers MS7 were (6-FAM) 5′-GTATTCCCCGTCTTGTCC-3′ forward and 5′-CTTTCTCCGTTCTTATTTCT-3′ reverse; and for MS10 were (NED) 5′-AAGTGTATTTTCCCGACG-3′ forward and 5′-CTTTTGCTTGCTCCGTTT-3′ reverse, respectively selected from a set of highly polymorphic markers with high heterozygosity in the Sri Lankan endemic populations with vivax malaria [13]. The core repeat sequences of the MS7 marker was (GAA)9 and that of the MS10 marker was GAA(GGA)_2_ AGA(GGA)_9_AGA(GGA)_4_AGAGGAAGA(GGA)_3_AGAGGAAGA(GGAAAA)_4_(GGA)_2_(AGA)_11_(GGA)_3_(AGA)_2_GGAAGA(GGA)_2_ with size ranges of amplicons 133–160 for the former and 180–306 for the latter.

PCR was performed with a Genosys PCR thermocycler (MJ Research Model PTC-200, Genosys, Provo, UT, USA). Amplification was confirmed in a 2% agarose gel and PCR products were stored at 4 °C in the dark. The product size was resolved by capillary electrophoresis in an ABI Prism 3130 Genetic Analyzer (Applied Biosystems), using GS500 LIZ as the internal size standard and analysed by GeneMapper software (Applied Biosystems). The electropherograms were scored for peaks above a cut-off of 300 relative fluorescent units for true amplification products. Alleles were characterized by the repeat length.

### Estimation of PQ and CPQ concentrations

**HPLC method** Quantification of the plasma PQ and CPQ levels among all the study subjects during steady state (on 7th day after the initiation of PQ) was performed using high-performance liquid chromatography (HPLC). Median plasma PQ and CPQ levels at day 7 were compared among patients of both the groups.

### Optimized chromatographic conditions

**Fig. 1:**
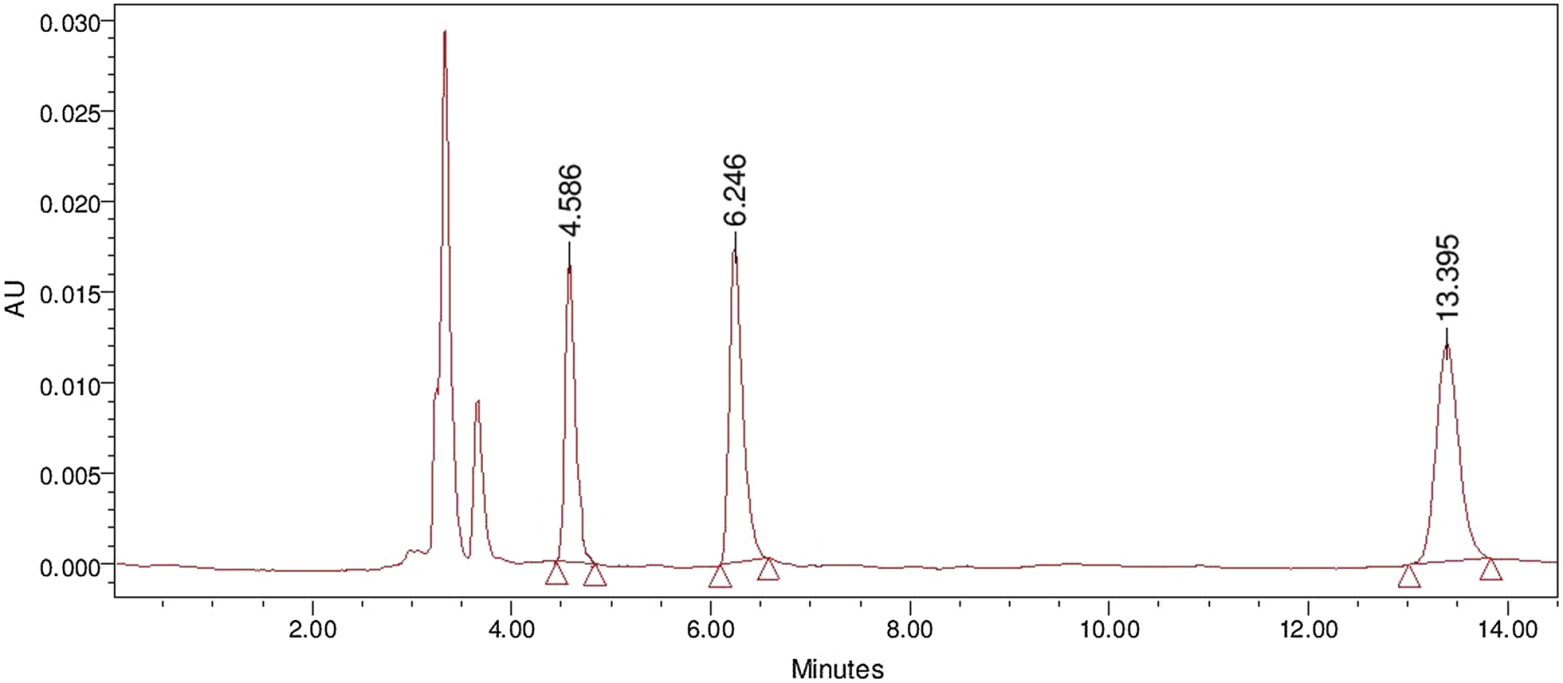
Chromatogram for quinine sulfate, primaquine and carboxyprimaquine

In the present study, a reversed phase HPLC (RP-HPLC) method was developed for the estimation of PQ and CPQ using quinine sulfate (QS) as an internal standard (ISTD) with wide linearity range of 150 ng/ml to 16 µg/ml. Separation of PQ, CPQ and QS was achieved on Waters^®^ separation module 2695 by applying isocratic reverse phase mode on Gracesmart RP C_18_ column (250 mm × 4.6 mm, 5 µ), using acetonitrile and 10 mM ammonium formate (0.2% triethyl amine) buffer pH adjusted to 3.0 with formic acid (30:70,% v/v) as mobile phase, at a flow rate of 1.0 ml/min, and drug molecules were detected at 265 nm on Waters^®^ dual absorbance detector 2487. The column temperature was maintained at 38 °C and auto sampler at 4 °C, retention time of QS, PQ and CPQ were observed at 4.58, 6.24 and 13.39 min, respectively. The run time was 15 min. Representative chromatogram is shown in Fig. 1.

### Optimized liquid–liquid extraction technique

To 180 µl of human plasma, 20 µl of 10 µg/ml QS solution and 20 µl of working stock of PQ and CPQ were added and vortexed for 2 min. One-thousand micro litre of *tert*butyl-methyl-ether and dichloromethane (2:1,  % v/v) were added and vortexed for 10 min for extraction of drug molecules to organic phase. Samples were centrifuged at 10,000 RPM for 15 min at 4 °C using cooling centrifuge to separate proteins from the organic phase. The resulted supernatant was collected and evaporated to dryness using nitrogen for 10 min at 40 °C under 10 kpa pressure. Finally, the dried residue was reconstituted in 100 µl of methanol, and vortexed for 1 min. This solution was transferred to total recovery vial and 40 µl of solution was injected into HPLC for analysis [14].

### Ethics statement

The ethics committee of Manipal Academy of Higher Education, (MUEC/021/2016-17) Manipal, India approved the study protocol. A signed written informed consent was obtained from each subject prior to enrolment. If the study subject was illiterate, an impartial third party witnessed the informed consent process. All subjects were informed the nature of the trial and that they were free to withdraw consent to participate at any time. The investigators and study personnel ensured confidentiality of all records.

### Data analysis

Frequency and percentage was used for categorical variable, mean and standard deviation or median and interquartile range was used to analyse continuous variable. Chi square test used to test association between two categorical variables. Independent sample *t* test was used to compare means across two groups; Mann–Whitney test was used to compare median across two groups. Data analysis was carried-out by SPSS version 15.

## Results

**Fig. 2:**
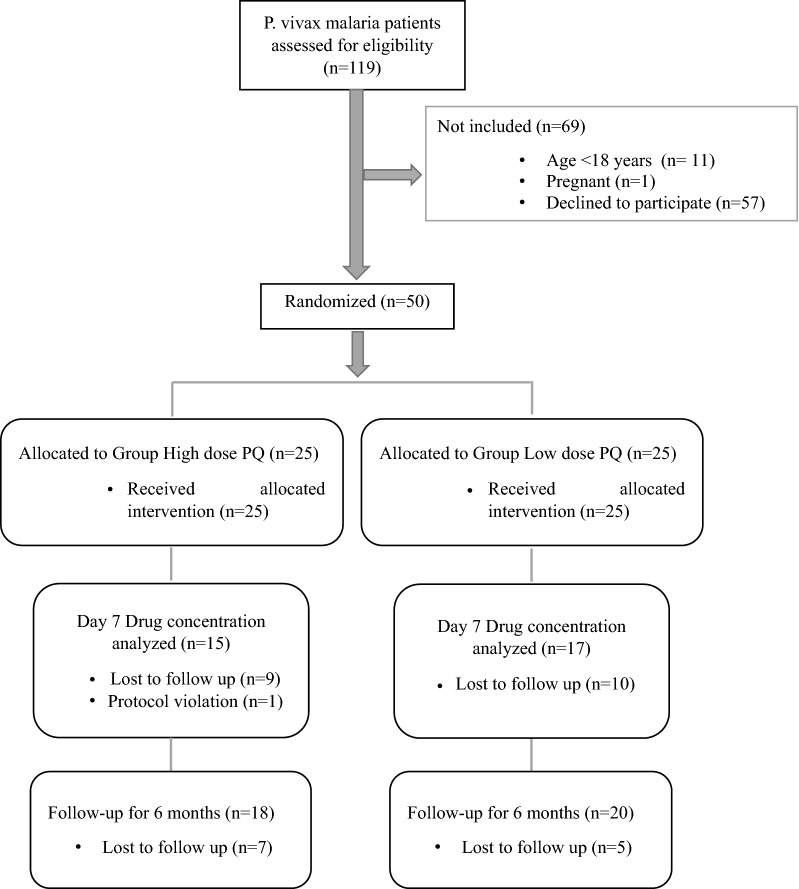
CONSORT flow diagram

During the recruitment period, 119 individuals were diagnosed with *P. vivax* by microscopy at the two study hospitals (Fig. 2). The main reason for not including patients was if they were unwilling to complete the lengthy follow up (57, 47.9%). Fifty participants were enrolled and randomized (1:1) to one of the two study arms, of whom 30 (60%) were available for the day 7 follow up for PQ and CPQ estimation A total of 38 participants completed 6 months’ follow-up. Only one protocol deviation occurred in which the participant reported 5 h late for the day 7 follow up drug concentration estimation. The study cohort included 45 (90%) male and 5 (10%) female participants. The mean (± SD) age of cohort was 42.0 (± 16.0) years, which ranged from 18 to 76 years. The baseline characteristics and laboratory features of the patients were similar among the treatment groups (Tables 1, 2). Half of the low dose PQ regimen subjects had a chronic disease, although this was not statistically significant.

**Table 1:** Demographic characteristics and outcomes of two groups

Characteristic	PQ high dose (N = 25) n (%) or Mean ± SD	PQ low dose (N = 25) n (%) or Mean ± SD	*p*-value
Gender			
Male	23 (92)	22 (88)	0.63
Female	2 (8)	3 (12)	
Age, years	42.0 ± 16.0	40.0 ± 15.0	0.34
Body weight, kg	67.9 ± 14.1	64.6 ± 13.7	0.40
Body mass index, kg/m^2^	25.0 ± 4.0	24.6 ± 5.2	0.43
Past h/o malaria	6 (24)	11 (44)	0.13
Pre-morbidities			
Diabetes mellitus	2 (8)	4 (16)	0.38
Hypertension	2 (8)	5 (20)	0.22
Chronic kidney disease	0	1 (4)	0.31
Heart disease	0	2 (8)	0.15
Severity			
Non-severe	24 (96)	24 (96)	1.00
Severe	1 (4)	1 (4)	
Schizonticidal treatment			
Chloroquine	20 (80)	19 (76)	0.73
Artemisinin combination therapy	5 (20)	6 (24)	
Use of personal protective measures during follow-up			
Mosquito repellents	16 (64)	18 (72)	0.54
Regular bed net	3 (12)	2 (8)	0.63
Study outcome			
Recurrence	2 (4)	2 (4)	–

**Table 2:** Clinical and laboratory features of two groups

	PQ high dose (N = 25) n (%) or Mean ± SD or Median (IQR)	PQ low dose (N = 25) n (%) or Mean ± SD or Median (IQR)	*p*-value
Fever	25 (100)	25 (100)	
Splenomegaly	3 (12)	2 (8)	0.63
Hepatomegaly	2 (8)	2 (8)	1.00
Haemoglobin, gm/dl	13.2 ± 1.7	13.5 ± 1.7	0.45
Urea, mg/dl	33.3 ± 19.9	31.7 ± 13.4	0.43
Creatinine, mg/dl	1.2 ± 0.4	1.1 ± 0.4	0.36
Total bilirubin, mg/dl	1.4 (1.3, 1.9)	1.5 (0.95, 3.0)	0.36
Direct bilirubin, mg/dl	0.6 (0.3, 0.8)	0.6 (0.3, 1.4)	0.12
Aspartate amino transferase, IU/l	39.5 (35.5, 54.0)	30.0 (27.0, 41.0)	0.38
Alanine amino transferase, IU/l	40.5 (29.0, 67.0)	34.0 (24.0, 44.0)	0.67
Alkaline phosphatase, IU/l	87.0 (62.5, 118.5)	82.0 (57.0, 110.0)	0.34
PQ concentration (µg/ml) (N = 30)	0.576 (0.186, 0.782)	0.184 (0.131, 0.282)	0.193
Carboxy PQ concentration (µg/ml) (N = 30)	2.401 (1.535, 4.119)	2.809 (0.817, 6.658)	0.945

One patient in each treatment arm had severe malaria. Both regimens were well tolerated. Adherence to the prescribed regimen was complete in 30 (60%) who were available for the day 7 follow up. No significant drug related adverse effects were noted among either study cohort.

In the current study, a sensitive, precise, accurate, reproducible and economic RP-HPLC was developed. QS was selected as the internal standard owing to its structural similarity and comparable pKa with PQ and CPQ. This optimized chromatographic method was validated as per US FDA bioanalytical method validation guidelines. Overall precision and accuracy were found to be well within the acceptance range (i.e., 85–115%) and lower limit of quantification was found to be 150 ng/ml. Extraction efficiency of the optimized method was found to be 75–91%. Stability of PQ and CPQ in plasma was performed by conducting long-term stability studies for 30 days, where degradation was found to be less than 2.0%. The optimized method was also validated for carry-over effect, system suitability, specificity, and sensitivity.

**Fig. 3:**
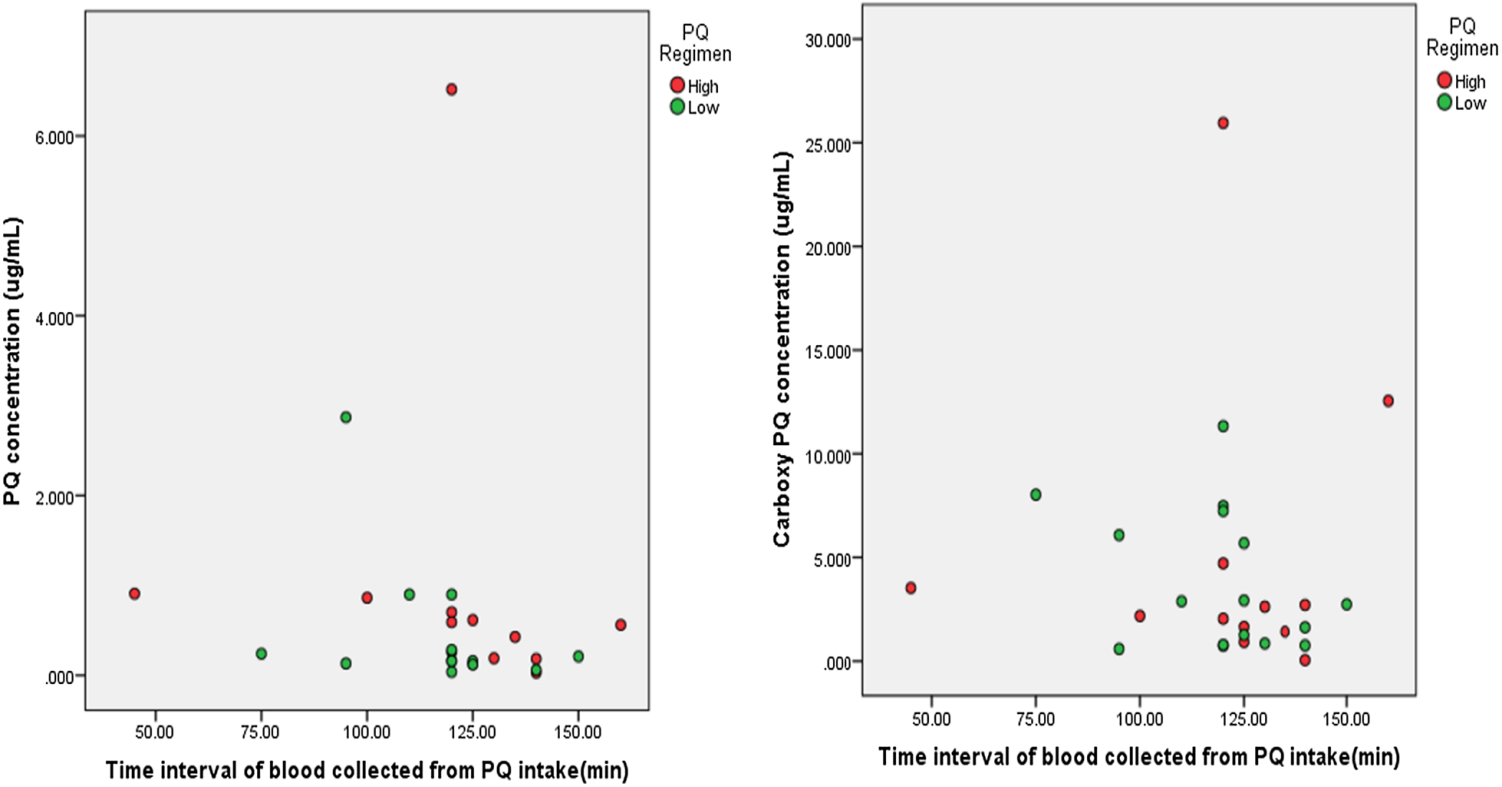
Plasma concentrations of primaquine and carboxyprimaquine. Scatter plot depicting the plasma concentration of PQ and CPQ plotted against time since PQ intake. Red circle indicates subjects prescribed with 0.5 mg/kg/day of PQ. Green circle indicates subjects prescribed with 0.25 mg/kg/day of PQ. There are two people with extreme observations on PQ and CPQ concentration. Their respective values are 6.5 µg/ml and 25.95 µg/ml

The plasma concentrations of PQ and CPQ has been depicted in Fig. 3. Median concentrations of PQ and CPQ on day 7 were not significantly different amongst the two groups (Table 2). The patient who was sampled after 5 h for the 7th day blood sample was excluded from the analysis.

### Recurrence

**Fig. 4:**
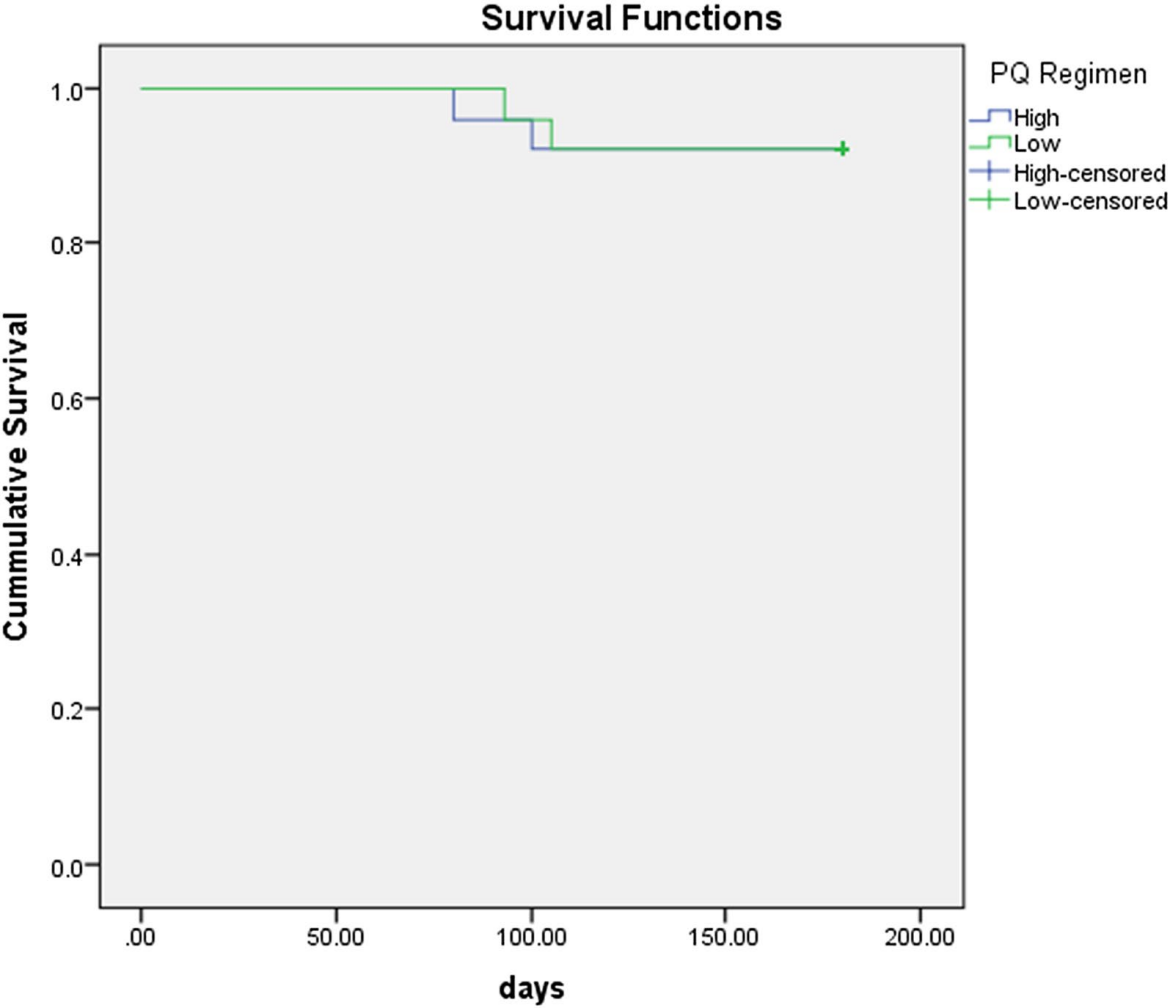
Kaplan Meir survival curve of time to recurrence across two groups

Recurrent infections occurred in 4 [8%] patients included in the present study, 2 patients in each arm. (Tables 1, 3). All presented with uncomplicated malaria. The intervals from starting the initial treatment to recurrence ranged from 80 to 105 days. Survival analysis with time to recurrence among the two regimens has been depicted in Fig. 4.

**Table 3:** Clinical characteristics, drug concentrations and genotyping of *Plasmodium vivax* among patients with recurrences

	Patient 1	Patient 2	Patient 3	Patient 4
Age (years)	22	57	20	60
Gender	Male	Male	Male	Male
Weight (kg)	84.7	83	70	60
Body mass index	28.40	29.76	25.40	23.73
Past h/o malaria	Yes	No	Yes	No
Clinical phenotype of index episode	Non-severe	Non-severe	Non-severe	Non-severe
Schizonticidal drug	Chloroquine	Artesunate + Doxycycline	Chloroquine	Chloroquine
Regimen of PQ	High	Low	Low	High
Actual dose of PQ received (mg/day)	45.0	22.5	15.0	30.0
Actual dose of PQ received (mg/kg)	0.53	0.27	0.21	0.50
Time to first recurrence (days)	105	100	93	80
Clinical phenotype of recurrent episode	Non-severe	Non-severe	Non-severe	Non-severe
Schizonticidal drug of recurrence	Chloroquine	Chloroquine	Chloroquine	Chloroquine
PQ dose during recurrent episode (mg/day)	45.0	15.0	37.5	30.0
Drug levels during index episode				
PQ Level (µg/ml)	6.518	0.240	0.159	NA
CPQ Level (µg/ml)	4.714	8.026	0.746	NA
Drug levels during recurrence				
PQ Level (µg/ml)	0.267	NA	0.399	NA
CPQ Level (µg/ml)	3.873	NA	0.536	NA
Amplicon sizes (MS7)				
Initial infection	342	351	351	345
Recurrent infection	342	351	351	345
Amplicon sizes (MS10)				
Initial infection	263	254	290	290
Recurrent infection	263	254	290	290

*NA* not available

### Genotyping of *Plasmodium vivax* to differentiate relapse from re-infection

**Fig. 5:**
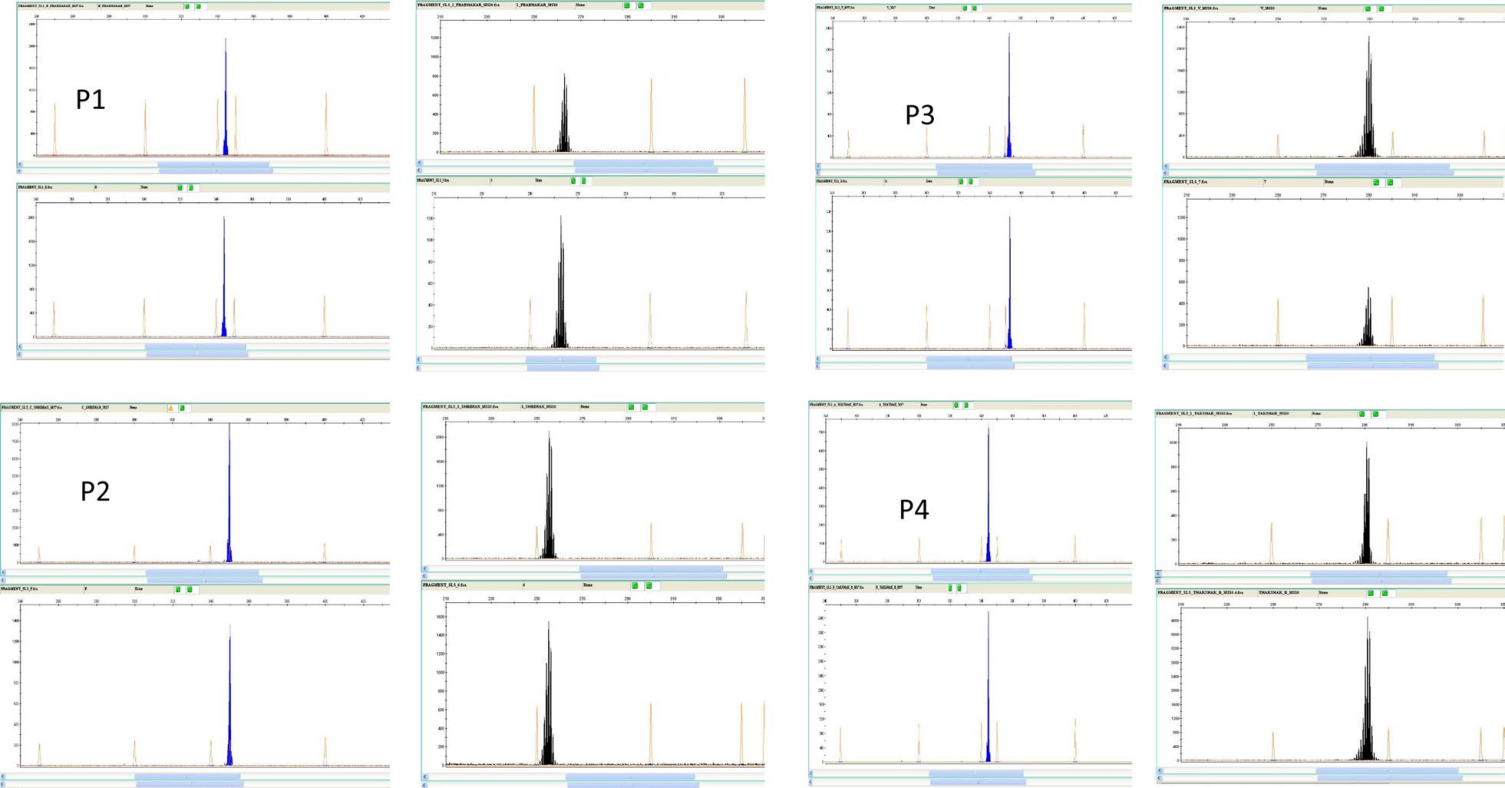
Four panels (P1 to P4) showing the results of fragment length of *Plasmodium vivax* DNA microsatellite markers MS7 and MS10 from capillary electrophoresis after polymerase chain reaction with fluorescent labelled (6-Fam and Ned) primers, from the four cases studied. Top row of each panel represents samples collected at initial infection and the bottom row at the time of reporting relapse of the disease

Genotyping suggested all four recurrences were homologous (isogenic) relapses. All four primary infections were of a single genotype and were different on microsatellite typing, but all four recurrences had the same alleles as the primary infections. Results of the capillary fragment length analysis of the MS7 and MS10 microsatellite markers from patients with recurrent malaria are depicted in Fig. 5 and Table 3. All were treated with CQ followed by PQ of 0.5 mg/kg for 14 day. Characteristics of patients with relapses are in Table 3.

## Discussion

This pilot study assessed the tolerability, safety and effectiveness of two radical cure PQ dosage regimens (0.25 mg/kg/day, 0.5 mg/kg/day up to 14 days). Both proved very effective. PQ is readily absorbed from the gastrointestinal tract, undergoes high first pass metabolism and is extensively distributed into body tissues. The plasma concentration peaks within 1–3 h after ingestion. PQ has a plasma elimination half-life of 4–9 h in healthy adults [15]. The metabolism of PQ is complex. A large number of metabolites have been identified. There are two main pathways, one via CYP450 2D6 and other CYP enzymes to reactive intermediates which are thought to mediate antimalarial activity and also haemolytic toxicity, and the other (via monoamine oxidase) to the biologically inert CPQ. CPQ is much more slowly eliminated than the parent compound with an elimination half-life of 22–30 h in healthy adults and so it is a potentially useful measure of adherence [16].

The present cohort consisted predominantly males; the gender imbalance is typical of patient population in the study setting and that of many others in malaria endemic areas of Asia [10, 17–19]. Although radical curative efficacy of observed PQ is usually over 90%, it is widely thought that adherence is poor to the 14 day regimens currently recommended. This small pilot study indicates that with appropriate explanation to the patients the effectiveness is good—in this series ~ 89%. It is highly likely that the four recurrences resulted from relapses as each pair of acute and recurrent isolates had the same microsatellite genotype but each differed from the other [20, 21]. Of the three relapses which had drug level measurement, one had very low day 7 CPQ concentrations strongly suggesting that the subsequent relapse resulted from poor adherence. One patient with relapse had high concentration of PQ during the index episode as well as during the recurrence, which may be suggestive of poor metabolizer phenotype. The prevalence of CYP2D6 poor metabolizers in this area is not known, but even if this is low, this small study suggests good PQ susceptibility. There was no difference between the high and low dose regimens of PQ in therapeutic effectiveness supporting previous studies from the Indian sub-continent and South America [7, 22–24]. A total dose of 3.5 mg base/kg seems sufficient for maximum hypnozoitocidal effects outside East Asia and Oceania. *Plasmodium vivax* cases recorded at health facilities during the non-transmission season likely arise from reactivation of dormant liver-stages. In the transmission season *P. vivax* cases are presumed to be arising both from new mosquito infections and relaps. [25]. The interval to relapse ranged between 80 and 105 days in this study which is longer than usual in East Asia, but is consistent with some studies from India which suggest an intermediate duration of latency [26–28].

This was a small study, and one-third of recruited patients did not complete the full 6 months follow up. This comparison was, therefore, underpowered to show significant differences between the two treatment arms. Nevertheless the effectiveness results do support the current dosing recommendation of PQ 3.5 mg base/kg total dose radical cure regimen in South India.

## Conclusion

The recurrence rate of vivax malaria in this study was 8% at 6 months follow-up and it occurred equally in high dose as well as low dose PQ regimens. Recurrence cases were of non-severe phenotype and genotyping suggested all four recurrences were homologous (isogenic) relapses. This small pilot trial supports the effectiveness of the currently recommended lower dose (0.25 mg/kg/day) 14 day PQ regimen for the radical cure of vivax malaria in South India.

## Abbreviations

PQ: primaquine
CPQ: carboxyprimaquine
PCR: polymerase chain reaction
WHO: World Health Organization
CQ: chloroquine
ACT: artemisinin-based combination therapy
HPLC: high-performance liquid chromatography
QS: quinine sulfate
ISTD: internal standard
RP-HPLC: reversed phase-high-performance liquid chromatography
IQR: interquartile range
MS: microsatellite
USFDA: United States Food and Drug Administration
